# Cultivating green workforce: The roles of green shared vision and green organizational identity

**DOI:** 10.3389/fpsyg.2023.1041654

**Published:** 2023-03-15

**Authors:** Xuejun Ma, Hasnain Bashir, Arslan Ayub

**Affiliations:** ^1^School of Economics and Management, Weifang University of Science and Technology, Weifang, Shandong, China; ^2^School of Law and Justice, Faculty of Business, Government and Law, University of Canberra, Canberra, ACT, Australia; ^3^National School of Management Studies, The University of Faisalabad, Faisalabad, Pakistan

**Keywords:** green talent management, green organizational identity, employee retention, green shared vision, social exchange theory

## Abstract

**Introduction:**

The current study anchors on the social identity theory (SIT) and social exchange theory (SET) to investigate the association between green talent management (GTM) and employee retention (ER), mediated by green organizational identity (GOI). Further, the study projects the moderator effect of green shared vision (GSV) in the direct association between GTM and GOI, and the indirect link between GTM and ER through the mediator effect of GOI.

**Methods:**

We collected time-lagged (i.e., three-wave) data from 495 frontline managers in the tourism service firms in Pakistan. Data are analyzed using SmartPLS SEM (V 3.3) to evaluate the measurement and structural models.

**Results:**

Our results support all the projected associations and confirm the direct relationships between GTM and ER (*β* = 0.480, CIs = 0.494, 0.578), GTM and GOI (*β* = 0.586, CIs = 0.517, 0.670), and GOI and ER (*β* = 0.492, CIs = 0.425, 0.566). The findings further reveal that GOI significantly mediates the relationship between GTM and ER (*β* = 0.257, CIs = 0.184, 0.312). In addition, the moderator effect of GSV significantly underpins the direct association between GTM and GOI (*β* = 0.512, CIs = 0.432, 0.587) and the indirect association between GTM and ER, mediated by GOI (*β* = 0.526, CIs = 0.441, 0.590).

**Discussion:**

This is the first study that explores a moderated mediation model to explain *when* and *how* tourism service firms can promote ER through inculcating GTM strategies. The findings indicate that service firms in the tourism industry must develop and retain green talent to exploit pro-environmental strategies.

## Introduction

Challenged by the escalating pressures of governmental programs to address global warming trepidations and embed green practices across business activities, service firms in the tourism industry face an imperative to cultivate and retain talent to address organizational sustainability and global climate change (Gardas et al., 2019; Ogbeibu et al., 2021). The global “war for talent” and “how to manage talent” remain significant challenges for service firms, particularly given that green talent is needed to help service firms to cultivate environmental sustainability (Song and Xie, 2019). An essential element in the research on employee retention (ER) is the need to retain green talented staff (Gardas et al., 2019). A massive stream of research has been conducted in exploring general talent management strategies that affect ER, *including* Ambrosius (2018), Baharin and Hanafi (2018), Gupta (2019), Narayanan et al. (2019), and Pandita and Ray (2018). However, only a few studies have explored green organizational strategies that can be linked with ER of green talent (Ogbeibu et al., 2021). Employee retention is defined as “keeping those members of staff that one wants to keep and not losing them from the organization for whatever reason, especially to the competitors” (Browell, 2003, p. 5).

This paper aims are to first examine the antecedent of ER with the primary focus on retaining green talent. In this milieu, we propose that green talent management (GTM) is a significant predictor that influences ER in tourism service firms. GTM or green soft talent management used interchangeably in this study, is defined as:

“a humanistic aspect of talent management that actively supports, and is committed toward, the development and retention of green talent by boosting talents’ commitment via effective communication, talent inclusiveness in decision-making process, organizational support for talent wellbeing and welfare, and effective and efficient leadership practices that inspire green talented team members to engender defined ecological initiatives for fostering environmental sustainability” (Ogbeibu et al., 2021, p. 1,474).

With an emphasis on the social exchange theory (SET, Blau, 1968), the present study contributes to the literature by predicting the association between GTM and ER. GTM, as an emerging concept, allows organizational leaders to advance green workplace initiatives by systematically attracting, nurturing, retaining, and deploying the right talent (Gardas et al., 2019). This corollary is based on the norms of reciprocity (Gouldner, 1960), which specifies that exchange relationships evolve when organizations take care of their employees (Narayanan et al., 2019). To the best of the authors’ knowledge, this is the first study that examines the impact of GTM on ER in tourism service firms.

Second, in addition to assessing the direct relationship between GTM and ER, this study explores a causal mechanism through which effective GTM strategies culminate into amplified ER. We draw on the social identity theory (Ashforth and Mael, 1989), and propose the mediating role of green organizational identity (GOI). GOI refers to “an interpretive scheme about environmental management and protection that members collectively construct in order to provide meaning to their behaviors” (Chen, 2011, p. 388). Investigating GOI as a causal mechanism may present opportunities to grasp a comprehensive rational framework of an organization that offers the maximum capacity to effect the activities of its members.

Third, to develop a more nuanced understanding of the GTM-retention nexus, this study explores the boundary effects of green shared vision (GSV) that might also underpin the relationship. GSV refers to “a clear and common strategic direction of collective environmental goals and aspirations that has been internalized by members of an organization” (Chen et al., 2015, p. 1171). According to Chen et al. (2015), GSV leverages organizations to inculcate green initiatives across all business activities and strategies. We propose that GSV moderates the relationship between GTM and ER linkage, mediated by GOI.

### Contributions of the study

The current study contributes to the extant knowledge on talent management in numerous ways. First, given an escalated gravity of interest in attracting and retaining talent in the 21st century organizations (Davern, 2021; Reis et al., 2021), characterized by increased accountability and responsibility toward environment (Saleh and Atan, 2021), organizations have increasingly put more emphasis on the sustainable talent management practices to foster environmental sustainability (Gaikwad et al., 2016; Jooss et al., 2021; Mitosis et al., 2021). This is in line with the increased governmental pressures to mitigate organizational impacts in global warming and surging instability of the 4^th^ “industrial revolution” (Ogbeibu et al., 2021). In this regard, existing talent management practices with an overlooked emphasis on human capital sustainability offer limited explanation to meet the global sustainable development goals (Saleh and Atan, 2021). As a result, the HR practitioners warrant updating existing talent management practices by instilling “green human capital development programs” (also GTM) in order to deploy and retain the right talent that may advance workplace green initiatives forward (Malik et al., 2021). This turns out to be our second contribution to the general talent management literature.

Ogbeibu et al. (2021) argued that GTM, emerged as an organizational strategy, facilitates organizational sustainability goals through the systematic attraction, nurture, and retention of green talent. Further, Gardas et al. (2019) encapsulated GTM under the umbrella of “Sustainable Human Resource Management” (SHRM), which constitutes two pillars, i.e., “sustainability of individuals,” and “sustainability of organizations.” The prime focus of this study is on the GTM as a mean to nourish ER. In order to address inflating environmental issues, research studies are on a surge in the recent years that cast GTM as a valuable factor in influencing a wide array of individual (Malik et al., 2021; Ogbeibu et al., 2021) as well as organizational factors (Guillot-Soulez et al., 2022). Besides, related research streams focusing on sustainability have studied green recruitment (Pham and Paillé, 2020), green business practices (Sarkar et al., 2020), green human resource management (Paulet et al., 2021), green jobs creation (Sulich and Sołoducho-Pelc, 2022), as a mean to foster environmentalism through green workforce. The current study, thus, advances the research stream of sustainable human resource management by predicting GTM as a plausible factor influencing ER.

Third, our study advances the existing debate on talent management and ER by predicting GOI as the mediating variable between GTM and ER. In particular, it suggests that employees with high levels of GOI may be more capable of translating GTM into enhanced ER, which not only influences their future employment prospects but also their current commitment levels and attachment to their jobs. Last but not the least, our study proposes the moderator effect of GSV in the direct association between GTM and GOI, and the indirect association between GTM and ER, mediated by GOI. By investigating GOI as the intervening variable, this research enriches the understanding of the boundary conditions of the GTM–ER link, mediated by GOI, that is under what conditions the associations are more or less likely to pronounce. To the best of author’s knowledge, this is the first study that examines the boundary conditions of GTM–ER link by projecting the mediating role of GOI and the moderating role of GSV.

The remainder of this study explains the link among the study variables (hypotheses development), followed by a justification of the research design, data collection and analysis, and discussion of the implications of the findings.

## Literature review and hypotheses development

### Relationship between GTM, GOI, and ER

Given talent management’s critical role in ER, many prior studies have examined the talent management-retention link (Hughes and Rog, 2008; Chami-Malaeb and Garavan, 2013; Pandita and Ray, 2018; Narayanan et al., 2019). Despite eloquent findings obtained by previous studies, some critical questions still need to be addressed yet about *when* and *how* talent management acts to predict ER (Chami-Malaeb and Garavan, 2013; Narayanan et al., 2019). Correspondingly, the concept of GTM warrants considerable empirical scrutiny (Gardas et al., 2019; Ogbeibu et al., 2021) that might answer how GTM translate into enhanced ER.

According to SET (Blau, 1968), employees’ attitudes and behavior depend on the exchange relationship with their organizations. When they perceive that their organizations invest in them, they feel a reciprocal obligation to exhibit positive attitudes and elevated performance (Narayanan et al., 2019). According to Dries and De Gieter (2014), organizations set tone for the exchange relationship with its employees through the selection in the talent pool. Narayanan et al. (2019) further corroborated that employees who develop positive feelings about their organizations achieve bigger performance targets, manifest dedication and intention to stay. This is because talent management (TM) is premised on finding and engaging the right person in the key positions, therefore, a superior performance is assured (Collings and Mellahi, 2009). It is argued that while determining attitudes, researchers should adopt a multidimensional aspect of attitude, which includes “cognitive,” “affective,” and “conative/behavioral” components (Ajzen, 1989). Among several attitudes that employees exhibit, the staying intention could be viewed as “conative/behavioral” component of employee attitude toward organization that practices TM (Narayanan et al., 2019). Thus, the intention to stay can predict ER (Narayanan et al., 2019). Besides, Chami-malaeb and Garavan (2013) endorsed that SET can provide a compelling reason for how TM translates into enhanced ER.

GTM, in a similar way, determines employees’ intention to stay in the organization that practices GTM (Ogbeibu et al., 2021). In GTM, “climate action initiatives tend to be driven *via* a conducive work environment, adhocracy organizational culture and effective provision of relevant resources” (Berraies et al., 2020), thus, leveraging employees to demonstrate sustainable environmental behavior (Kalyar et al., 2021). Moreover, the findings from previous studies indicate that GTM is negatively related to the turnover intention of employees (Du Plessis et al., 2015; Barkhuizen and Schutte, 2017; Abdul et al., 2019). On contrary, Ogbeibu et al. (2021) investigated the impact of green hard and soft TM on turnover intention and found a positive association among them. The mixed findings from the past literature advocate further investigation of the phenomenon (Ogbeibu et al., 2021). Drawing on SET, we propose that GTM significantly predicts ER in service organizations. Therefore,

*H1*. GTM is positively linked with ER.

The discussion of organizational identity is an extension of social identity theory, which describes social identity as “that part of an individual’s self-concept which derives from his knowledge of his membership of a social group (or groups) together with the emotional significance attached to that membership” (Tajfel, 1978, p. 6). The concept of “organizational identity” is concerned with “collective identity” (Albert and Whetten, 1985). Mael and Ashforth (1995) defined organizational identification as “a perceived oneness with the organization and the experience of the organization’s successes and failures as one’s own; it generates self-referential or self-defining beliefs about one’s organization.” The construction of organizational identity is facilitated through symbols and language (Corley et al., 2006). According to Corley et al. (2006), organizational identity serves as a “metaphor” for describing organizations or an actual phenomenon and influences the actions of organizational members in fulfilling organizational goals.

Addressing the GTM strategy, GOI is subjected to a positive and distinct identity that strengthens the employee-employer bondage (Schlager et al., 2011). Earlier research on socially responsible human resource management practices implies that employees internalize organizational values when they perceive that their organization manifests environmental concerns (Newman et al., 2016). The inducement of such environmental practices pervades a sense of pride among employees (Hassan, 2007). Particularly, GTM practices result in increased levels of GOI. That is, “when concern for the environment becomes an integral component of organizational identity, environmental issues become harder to ignore” (Weick, 1988; Chen, 2011). Besides, social identity theory implies that employees develop a high sense of self-esteem due to the perception of their membership with a reputed organization that demonstrates environment-related concerns (Ashforth and Mael, 1989; Newman et al., 2016). By exploiting the GTM, organizations become more capable of elevating their positive image and reputation among its stakeholders as its employees are dedicatedly involved in the eco-friendly activities that benefit the external stakeholders. Hence, they feel proud to be a member of that organization (Bauman and Skitka, 2012), thus, inflating GOI. Therefore,

*H2*. GTM is positively linked with GOI.

In consolidation, we also suggest a mediating role of GOI, such that employees’ perception of their membership with an organization that manifests GTM practices enhances their likelihood of staying in the organization. The study proposes that employees with higher levels of GOI will more likely translate into enhanced ER. This is consistent with the social identity theory and SET, employees who perceive that they belong to an organization that shows concern for its stakeholders (Ogbeibu et al., 2021), and inculcate GTM practices to cultivate environmental sustainability; feel a reciprocal obligation to exhibit eco-friendly behaviors (Blau, 1968). Hence, GOI is a causal mechanism that translates GTM practices into increased ER. A host of recent research has found GOI’s significant mediating role in the relationship between green innovation strategy and green creativity and innovation (Song and Yu, 2018). In short, the perception of GOI underpins the underlying GTM-ER nexus. Therefore,

*H3*. GOI is positively linked with ER.

*H4*. GOI mediates the relationship between GTM and ER.

### The moderating role of GSV

**Figure 1:**
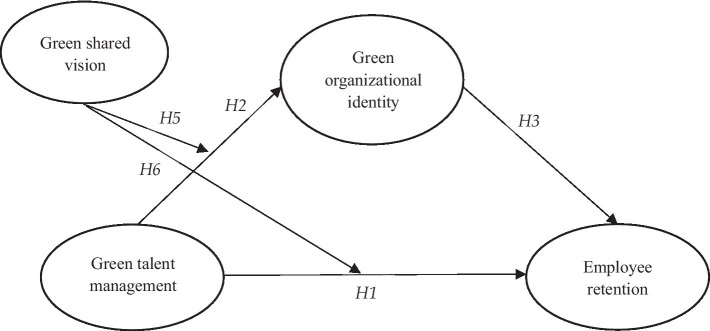
Conceptual model.

Although we generally expect a positive link between GTM and GOI (and ER), the literature’s variability in the relationship between GTM and employees’ staying intention suggests the potential for moderators (Figure 1). We, thereby, expect a moderating effect of GSV on the relationship between (1) GTM and GOI and (2) GTM and ER through the mediating role of GOI. According to the social identity theory, GSV is a catalyst that facilitates the development of GOI because it provides a collective strategic direction that can navigate members’ actions toward environmentalism (Chen et al., 2015). Furthermore, Mackie and Goethals (1987) argued that shared vision offers a common “strategic direction” that can divulge convergent goals.

According to Aragón-Correa (1998), an organization leverages shared vision by communicating the firm’s goals to members and sharing the obligation to accomplish organizational objectives. Similarly, Pearce and Ensley (2004) contended that shared vision allows organizations to provoke desired conduct in members, thus ensuring the convergence toward long-term goals. However, “if managers fail to share their goals, visions may become purely rhetorical, resulting in disillusionment and distrust instead of inspiration and motivation” (Oswald et al., 1994, p. 479). Shared vision enhances improved environment-friendly initiatives through a repertoire of mutual interest and vision (Hart, 1995) through inducing GTM. Nevertheless, empirical research is scant in linking shared vision to adopting proactive environmental strategies (Alt et al., 2015), particularly concerning GTM. In addition, the authors advocated that the implications of shared vision extend beyond developing proactive environmental strategies (i.e., GTM) (Alt et al., 2015). Employees view their contributions as meaningful when they perceive their organizations indoctrinate GSV (García-Morales et al., 2011; Chen et al., 2015). Hence, they feel more comfortable in developing environmental strategies, establish a conjoint blueprint for forthcoming progresses, promulgate norms and values, and exceed performance marks (Chang et al., 2019).

Once proactive environmental strategies (i.e., GTM) are established, the extent to which these strategies will decipher into superior environmental performance will rely on the GSV between organization and its members, especially because it requires a high degree of employee involvement in its execution (Hart, 1995; Alt et al., 2015). GTM strategies entail change and innovation, which may not be viewed or welcomed as ineludibly significant by all internal stakeholders (Delgado-Ceballos et al., 2012). Divergence may ensue in the interpretation of such strategies due to diversity of interest among managers and employees (Calantone et al., 2002), departmental differences (Brown and Eisenhardt, 1995), and function “myopia” (Sinkula et al., 1997). This may undermine the implementation of GTM strategies, resultantly preventing firms’ responses to market trends or environmental shocks. However, a shared vision can influence strategy implementation by mitigating conflicting interests and ambiguities (Jansen et al., 2008), teams and departmental coordination (Calantone et al., 2002), and redefining organizational purpose (Real et al., 2014). Thus, GSV serves as a stimulating variable that strengthens the association between GTM and GOI such that at high levels of GSV, the relationship is more potent than at low levels of green of shared vision. Therefore,

*H5*. GSV moderates the association between GTM and GOI, such that the relationship is strong (weak) at high (low) levels of GSV.

Cumulatively, the above projections suggest a moderated mediation model (Sahabuddin et al., 2021). As argued above, GSV moderates the relationship between GTM and GOI. Hence, this engagement focusing on SET, in turn, predicts ER. We, therefore, submit that GSV intervenes in the indirect relationship between GTM and ER, mediated by GOI. Therefore,

*H6*. GSV moderates the association between GTM and ER, such that the relationship is strong (weak) at high (low) levels of GSV.

## Methods

### Sample and procedure

The study gathered data by employing a time-lagged (i.e., “three-wave”) research design from service employees in the tourism firms in Pakistan. The data were gathered in three waves with a time interval of eight weeks between each wave. This helped the researchers to minimize the possible biases that may arise due to the causal effects of the mediation mechanism that should be tapped over a period of time (Maxwell and Cole, 2007). However, failure to do so may lead to possible biases in measuring the estimates (Cole and Maxwell, 2003).

The authors administered questionnaires to the selected firms in the tourism industry, and front line managers were approached to participate in the survey. The study collected data through a purposive sampling technique aimed to collect arbitrary responses from the target respondents (Saunders et al., 2009). The participants were given the questionnaires along with the cover letters that explained the study’s purpose. Further, they were assured about the confidentiality of their responses. It also detailed participants the request for their voluntarily participation in the survey, and they could discontinue participating in the survey without any reason if they want so. In addition, the cover letter contained the instructions to generate the key for each participant, e.g., using the first letter of their last name with their city codes.

As recommended by Hair et al. (2017), we observed the rule of thumb for the sample size consideration in SmartPLS SEM. According to the authors, the sample size should be equal to or greater than “10 times the maximum number of arrow heads pointing at the latent endogenous variables,” or “10 times the largest number of the structural path” (Hair et al., 2017, p. 48). Several other well-cited studies employing SmartPLS SEM observed the minimum sample size of 207 to be appropriate for measuring the structural paths (e.g., Hassan and Ayub, 2019; Sahabuddin et al., 2021; Yao et al., 2022).

In the first wave, 600 questionnaires were administered to gather data for GTM, GSV, and demographic details of the respondents. Of which, the participants returned 554 questionnaires. The authors assessed the questionnaires and eliminated 18 incomplete and/or wrongly filled questionnaires. After eight weeks, the authors contacted those 536 respondents and collected responses concerning GOI. The authors received 514 completely filled questionnaires, of which 495 accorded with the original responses. In the third wave, the authors contacted the 495 participants and gathered data about ER.

Finally, we combined all the responses collected in each wave through the key generated by each participant. We processed 495 completed questionnaires (“response rate” 83%). Of the total respondents, 59% were men, and 41% were women participated in the survey; the mean age was 37.68 years with a standard deviation of 5.41 years. With respect to occupation, 35 and 65% of the respondents were in “lower” and “middle” managerial positions, respectively. Concerning tenure, 14% of the respondents worked in their organizations for “six to 12 months,” 24% of the respondents worked for “1 year to 4 years,” 29% have worked for “4 to 7 years,” 17% have worked between “7 and 12 years,” and 16% have worked for “more than 12 years.” Also, 39% of the respondents worked for “public sector organizations,” and 61% were from “private sector firms.”

### Measures

We adopted established scales from previous studies and disseminated them in the English language, as English is a “medium of instruction” at the schools/colleges/universities levels and is used as an official language in the business sector in Pakistan. The research instruments were measured on a five-point Likert scale from 1 (“strongly disagree”) to 5 (“strongly agree”) (Appendix I).

### GTM

The instrument to measure GTM was adopted from Ogbeibu et al. (2021). Its measure includes seven items and the sample items are “my organization cares about my well-being and offers considerable support for my welfare when executing green centered initiatives,” and “my organization offers me a considerable degree of autonomy when carrying out green related tasks.”

### GOI

The instrument to measure GOI was adopted from Chen (2011). Its measure includes seven items and the sample items are “top managers, middle managers, and employees of the organization are proud of its history regarding environmental management and protection,” and “top managers, middle managers, and employees of the organization are knowledgeable about its environmental tradition and culture.”

### GSV

The instrument to measure GSV was adopted from Jansen et al. (2008). Its measure includes four items and the sample items are “there is commonality of environmental goals in the company,” and “all members in the company are committed to the environmental strategies of the company.”

### ER

The instrument to measure ER was adopted from Kyndt et al. (2009). Its measure includes 11 items and the sample items are “I’m planning of working for another company within a period of 3 years,” “it does not matter if I’m working for this company or another, as long as I have work,” and “I love working for this company.”

### Control variables

Following the previous studies, individual demographics, such as age, gender, occupation, and tenure, were taken as controlled variables (reported in Table 1).

**Table 1:** Effects on endogenous variables.

Hypotheses	*β*	CI (5, 95%)	SE	*t*-Value	*p*-Value	Decision	*f* ^2^	*R* ^2^	*Q* ^2^
Age^1^	0.014(*n.s.*)	(−0.042, 0.044)	0.023	0.532	0.405				
Gender^2^	0.069(*n.s.*)	(−0.010, 0.103)	0.022	0.468	0.345				
Occupation^3^	0.084(*n.s.*)	(−0.008, 0.128)	0.033	0.647	0.361				
Tenure^4^	0.025(*n.s.*)	(−0.040, 0.017)	0.019	0.457	0.456				
*H1* GTM →ER	0.480***	(0.494, 0.578)	0.072	8.582	0.000	Supported	0.343	0.496	0.343
*H2* GTM → GOI	0.586***	(0.517, 0.670)	0.051	7.234	0.000	Supported	0.181	0.614	0.534
*H3* GOI → ER	0.492***	(0.425,0.566)	0.038	10.342	0.000	Supported	0.321		
*H5* GTM × GSV → GOI	0.512***	(0.432, 0.587)	0.052	4.242	0.001	Supported	0.238		
*H6* GTM × GSV → ER	0.526***	(0.441, 0.590)	0.062	9.243	0.000	Supported	0.302		

GTM, green talent management; GOI, green organizational identity; GSV, green shared vision; ER, employee retention; ***significance *p* < 0.05 (1.96).

1 = control variables.

2 = control variables.

3 = control variables.

4 = control variables.

### Data analysis

The study analyzed data using “partial least square structural equation modeling” (PLS-SEM) through SmartPLS (v 3.3). We justify the use of PLS-SEM over CB-SEM based on the following reasons. First, the study’s objective is to assess the “predictive capability” of the structural paths, i.e., “to maximize explained variance in the latent endogenous variables rather than theory confirmation” (Hair et al., 2017). Besides, the study assessed a complex moderated mediation model, i.e., in addition to estimating the main effects, the study also assessed the moderator effect of GSV (Henseler and Fassot, 2010).

## Results

### Measurement model

The study assessed the “measurement model” using “internal consistency,” “convergent,” and “discriminant” validity criteria (Hair et al., 2017). For evaluating “internal consistency,” in addition to the “Cronbach’s alpha,” the study measured the “composite reliability” (CR) of constructs, as recommended by Hair et al. (2017). Table 2 indicates that all the values of “Cronbach’s alpha” and CR for GTM (α = 0.845, CR = 0.890), GOI (α = 0.876, CR = 0.901), GSV (α = 0.842, CR = 0.880), and ER (α = 0.826, CR = 0.874) are greater than the threshold value of 0.6 and 0.7 (Cronbach, 1951; Hair et al., 2017), thus, providing evidence of “internal consistency.” For the “convergent validity,” the authors evaluated “outer loadings” and AVE with the acceptable minimum threshold of 0.5 (Henseler et al., 2009; Chin, 2010). Results of the analysis show that all the values are above the minimum acceptable threshold, hence, ensuring “convergent validity” of the study.

**Table 2:** Validity and reliability for constructs.

	Loadings	AVE	CR	Cronbach’s alpha
Green talent management		0.582	0.890	0.845
GTM1	0.712			
GTM2	0.802			
GTM3	0.782			
GTM4	0.762			
GTM5	0.687			
GTM6	0.823			
GTM7	0.762			
Green organizational identity		0.551	0.901	0.876
GOI1	0.726			
GOI2	0.672			
GOI3	0.800			
GOI4	0.781			
GOI5	0.763			
GOI6	0.746			
GOI7	0.681			
Green shared vision		0.546	0.880	0.842
GSV1	0.664			
GSV2	0.803			
GSV3	0.751			
GSV4	0.732			
Employee retention		0.591	0.874	0.826
ER1	0.764			
ER2	0.782			
ER3	0.827			
ER4	0.784			
ER5	0.693			
ER6	0.810			
ER7	0.782			
ER8	0.694			
ER9	0.696			
ER10	0.790			
ER11	0.814			

GTM, green talent management; GOI, green organizational identity; GSV, green shared vision; ER, employee retention; AVE, average variance extracted; CR, composite reliability.

In addition, the study assessed the “discriminant validity” that reflects the degree to which a latent variable empirically differs from other latent variables (Hair et al., 2017). For assessing “discriminant validity,” the study examined the “Fornell-Larcker” criterion and “Heterotrait-Monotrait” (HTMT) ratio (Hair et al., 2017). Table 3 shows the results of “Fornell-Larcker.” In addition, HTMT ratio were assessed using “bias-corrected and accelerated” (BCa) “bootstrapping technique,” using a resample of 5,000 by employing a one-tailed *t*-test with 90% significance level (in order to warrant an error probability of 95%). Table 3 presents the HTMT ratio, all the values are lesser than the threshold value of HTMT_.85_, thus, ensuring the “discriminant validity” of the study (see Figure 1).

**Table 3:** Discriminate validity.

	Fornell-Larcker criterion				HTMT criterion			
	GTM	GOI	GSV	ER	GTM	GOI	GSV	ER
GTM	0.762							
GOI	0.672	0.742			0.773 CI._0.900_ [0.680;0.841]			
GSV	0.651	0.563	0.738		0.624 CI._0.900_ [0.546;0.712]	0.762 CI._0.900_ [0.695;0.847]		
ER	0.462	0.422	0.264	0.768	0.721 CI._0.900_ [0.633;0.792]	0.512 CI._0.900_ [0.440;0.584]	0.666 CI._0.900_ [0.608;0.742]	

GTM, green talent management; GOI, green organizational identity; GSV, green shared vision; ER, employee retention; CI, bootstrapping 90% confidence intervals (*n* = 5,000) (one-tailed).

### Structural model

Further, we evaluated the “structural model” by analyzing the path analysis to examine the study’s hypotheses. The study utilized a “non-parametric,” “bootstrapping procedure.” In addition to the “path coefficients,” we also assessed the “structural model” by employing the following criteria, such as “coefficient of determination” (*R^2^*), “predictive relevance” (*Q^2^*), and the “effect sizes” (*f^2^*). The value of “cross-validated redundancy” above 0 indicates the “predictive capability” of the model. In addition, we reported the effect sizes to ensure the “predictive accuracy” of the model. The results of the “structural model” are presented in Table 1. The analysis indicates that GTM has a significant positive association with ER (*β* = 0.480, *t* = 8.582, *p* = 0.000, *f^2^* = 0.343), supporting *H1*. Besides, GTM has a significant positive impact on GOI (*β* = 0.586, *t* = 7.234, *p* = 0.000, *f^2^* = 0.181), supporting *H2*. Moreover, GOI has a significant positive impact on ER (*β* = 0.492, *t* = 10.342, *p* = 0.000, *f^2^* = 0.321), supporting *H3*.

**Figure 2:**
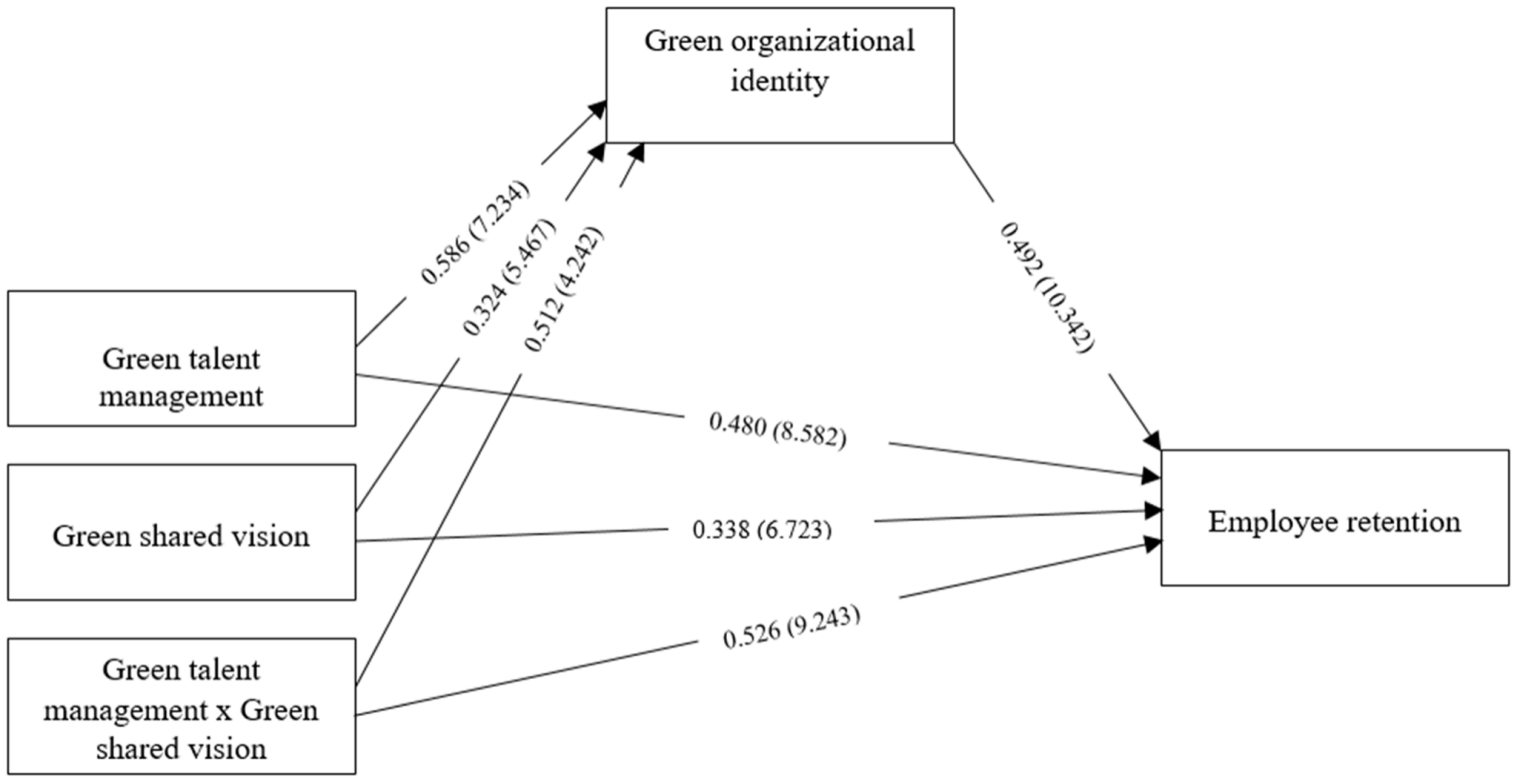
Structural equation model.

Moreover, the study employed a “two-stage approach” to examine the moderating effect (Hair et al., 2017). According to Henseler and Fassot (2010), the two-stage approach “exhibits a high level of statistical power,” as compared to orthogonal or product indicator approach. We measured the moderator effect size using BCa bootstrapping approach with a resample of 5,000. Table 1 shows that the interaction term (GTM_GSV) has a significant positive impact on GOI (*β* = 0.512, *t* = 4.242, *p* = 0.001, *f^2^* = 0.238), with medium effect size; and ER (*β* = 0.526, *t* = 9.243, *p* = 0.000, *f^2^* = 0.302), with large effect size, supporting *H5* and *H6*. The SEM is presented in Figure 2.

**Figure 3:**
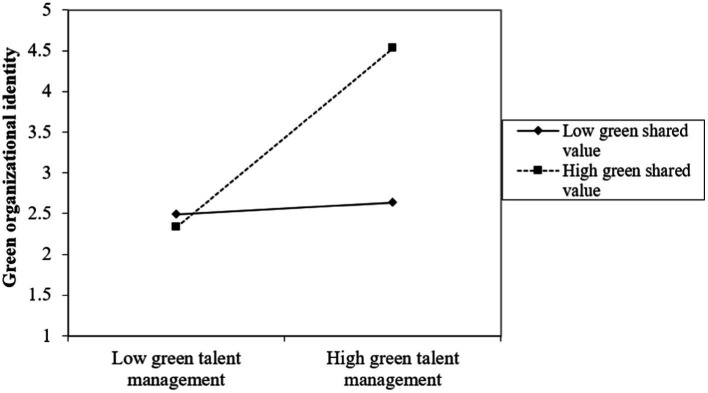
Interaction effect of green talent management and green shared vision on green organizational identity.

**Figure 4:**
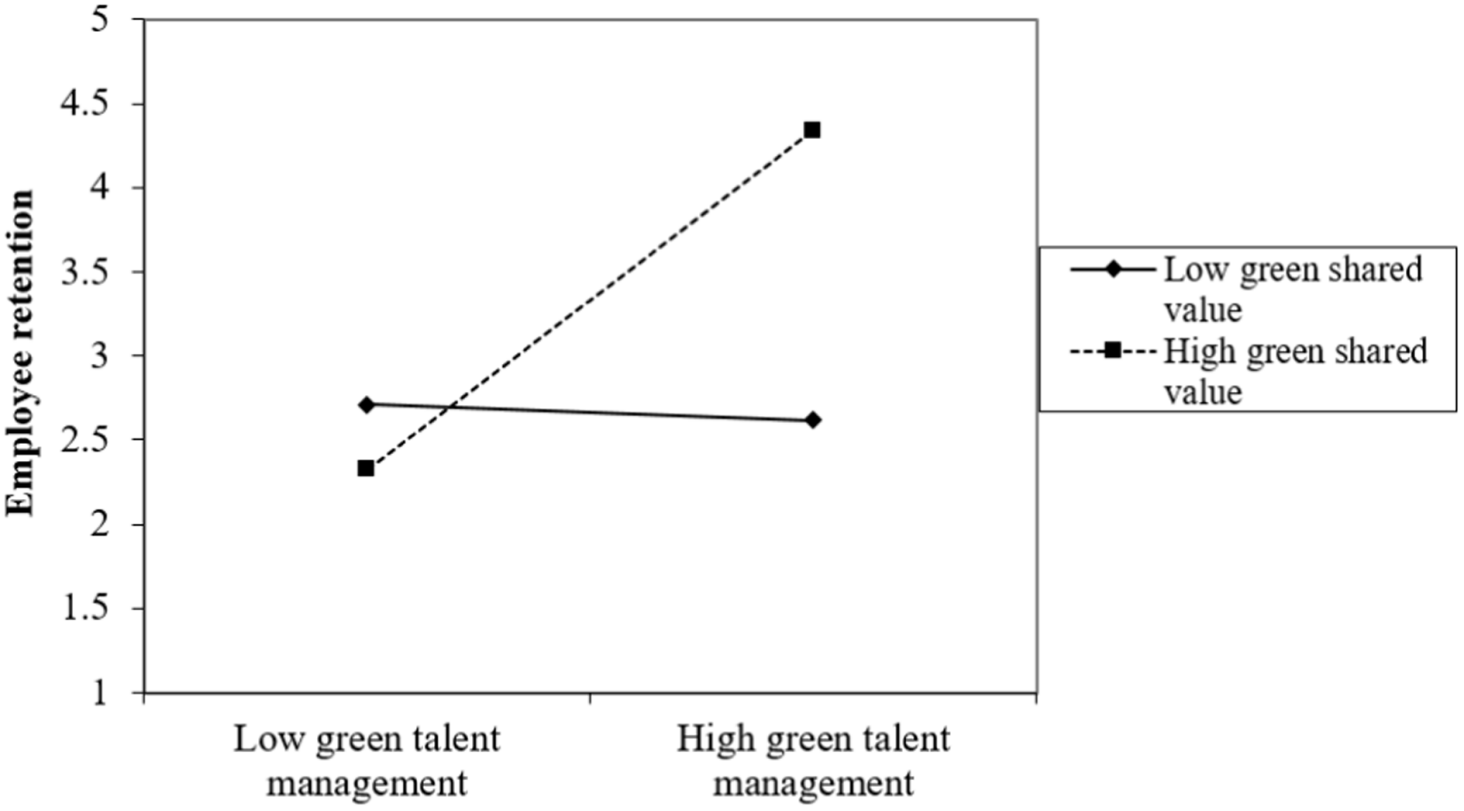
Interaction effect of green talent management and green shared vision on employee retention.

Further, we plotted a simple slope analysis to understand the interaction effect of GTM_GSV on GOI and ER (Dawson, 2014) (shown in Figures 3, 4). The simple slope analyses illustrate that at high levels of GSV the direct association between GTM and GOI and the indirect relationship between GTM and ER, mediated by GOI, is more pronounced than at low levels of GSV.

In addition, we also predicted the mediating role of GOI in the relationship between GTM and ER. In order to evaluate the mediation analysis, we adopted Zhao et al. (2010) “mediation approach.” We obtained point estimates of the indirect effect using BCa “bootstrapping technique” with a 5,000 resample (Hair et al., 2017). Table 4 shows that the total effect of GTM on ER is significant, with 95% CIs (0.657, 0.812) and the indirect effect of GTM on ER through the mediating role of GOI is significant, with 95% CIs (0.184, 0.312), indicating complementary mediation. In addition, we also assess “variance accounted for” (VAF) to assess the mediation analysis. The rule of thumb for VAF is as follows: <20% indicates “no mediation,” between 20–80% indicates “partial mediation,” >80% indicates full mediation (Nitzl et al., 2016). VAF value of 34.89% indicates that GOI partially mediates the relationship between GTM and ER, supporting *H4*.

**Table 4:** Summary of mediating effect tests.

	Path	*t*-Value	BCCI		Path	*t*-Value	95% BCCI	Decision	VAF
Total effect GTM → ER	0.737	9.536	(0.657, 0.812)	Indirect effect *H4* GTM → GOI →ER	0.257	4.121	(0.184, 0.312)	Supported	34.89%

GTM, green talent management; GOI, green organizational identity; GSV, green shared vision; ER, employee retention; VAF, variance accounted for (indirect effect/total effect*) *total effect: direct effect + indirect effect).

Furthermore, the study also measured the “goodness-of-fit index” (GFI), using Tenenhaus et al. (2005) diagnostic tool. The authors defined GFI as “the geometric mean of the average communality and average *R*^2^.” GFI results are shown in Table 5 with a value of 0.501 greater than minimum threshold of 0.36 for a large effect size of *R*^2^, ensuring a good model fit (Hoffmann and Birnbrich, 2012). Finally, we also examined the Stone-Geisser’s *Q*^2^ with an “omission distance” of 7. The analysis produced the value significantly greater than 0, thus establishing the model’s “predictive relevance.”

**Table 5:** Goodness-of-Fit Index (GFI).

Constructs	AVE	*R* ^2^
GTM	0.582	
GOI	0.551	0.534
GSV	0.546	
ER	0.591	0.343
Average scores	0.570	0.440
(*GFI =* $\sqrt{\bar{AVE}\times \bar{R^{2}}}$)	0.501	

AVE, average variance extracted; GTM, green talent management; GOI, green organizational identity; GSV, green shared vision; ER, employee retention.

## Discussion

Given the critical role of the tourism industry in ecosystem protection and development, developing and retaining green talent to enhance environmental sustainability has become an increasingly researched hotspot in environmental management (Gardas et al., 2019). Therefore, the chief contribution of this research is the theorization and evaluation of a hitherto unexplored moderated mediation model that may predict ER in the tourism industry. Hence, the study investigated the impact of GTM in determining ER through GOI’s mediating role and GSV’s moderating role. The study drew on social identity theory and SET to propose and test the theorized model, thus adding insights to environmentalism literature. As projected, the study found that GOI leverages meaning to members of an organization to implement environment-related strategies, translating into enhanced ER. Moreover, the study found the positive impact of the boundary effects of GSV on the underlying linkage. Specifically, the findings of this study support the theorized projections such that:

The study demonstrated that *H1* which states that GTM has a significant positive influence on ER, is supported. The results of this study advance the prior research on the link between GTM and turnover intention (Ogbeibu et al., 2021), by projecting that GTM facilitates ER in the tourism industry. Moreover, another justification of *H1* explain that GTM leverages meaningfulness in one’s job which serves as a mean to enhance ER. Further, we expect that our findings advance the impact of GTM on other employee outcomes, *such as* job satisfaction (Freire and Pieta, 2022) and engagement (Ababneh, 2021).

Similarly, the second hypothesis *H2* states that GTM has a significant positive influence on GOI. Extant research on organizational identification suggests that employees perceive that organizational identity dominates personal identity (Shah et al., 2021), and they view themselves as a larger whole. Therefore, GTM holds significant relevance in predicting GOI, which in turn, culminates into enhanced ER. This supports the hypothesis *H3*, which states that GOI mediates the link between GTM and ER. The result of this analysis is in harmony with prior research which found that organizational identification mediates the association between green human resource management and organizational citizenship behavior (Freire and Pieta, 2022).

In addition, the study predicted the moderating role of GSV in the association between GTM and ER, mediated by GOI (*H4* and *H5*). Our findings support the intervening role of GSV on the association between GTM and ER, mediated by GOI such that at high levels of GSV the relationships are more pronounced than at low levels of GSV. Our findings are similar to previous study on the moderating role GSV in the link between green HRM and green innovation. By investigating GSV, the study contributes to the literature of sustainable human resource management such as GSV infuses the internalization of workplace green initiatives by offering a mutual strategic direction that can divulge convergent goals, thereby, eliciting the association between GTM, GOI, and ER.

### Theoretical implications

The study adds several unique contributions to the existing literature on environmental strategies in the tourism industry by determining the linkage between GTM and ER. First, this is the first study that examined the impact of GTM on ER in the tourism industry. Although prior studies have identified the connection between TM and ER (Hughes and Rog, 2008; Chami-Malaeb and Garavan, 2013; Pandita and Ray, 2018; Narayanan et al., 2019), however, the association between GTM and ER has remained unexplored to date. Though, a host of research in recent years has investigated the impact of GTM on turnover intention of employees (Ogbeibu et al., 2021). However, their findings provide divergent acumens such as the authors found GTM as a predictor of turnover intention. In juxtapose the findings of this study sanction a substantial impact of GTM on ER. With a focus on SET (Blau, 1968), the study also contributes to the theoretical underpinning of SET by extending its applicability to the underlying GTM-ER nexus.

In addition, we proposed that GTM may determine ER; however, the process is interceded by a causal mechanism. We examined the mediating role of GOI and found that employees’ perception of environment-related organizational concerns leverages superior identity, which engenders GTM practices to achieve convergent goals. This turns out to be our second contribution such that by drawing on the social identity theory, we predicted that GOI offers maximum capacity to transform GTM strategies in the manifestation of eco-friendly business practices, ultimately enhancing employees’ intention to stay in an organization, based on the promulgation of a collective identity (Chang et al., 2019). The findings of this study are in harmony with preliminary research on GOI. For instance, Song and Yu (2018) found a partial mediation effect of GOI in the relationship between green innovation strategy and green creativity.

Third, the study extends the boundary conditions of the GTM-ER nexus. With an emphasis on the social identity theory and SET, we found that GSV stimulates the direct relationship between GTM and GOI and the indirect relationship between GTM and ER, mediated by GOI. Assessing the boundary effects of GSV allowed us to address the variability in the previous findings of the divergent impacts of GTM on ER (Ogbeibu et al., 2021). For instance, we hypothesized that differences in interests among managers and employees might limit the potential impact of GTM practices on GOI and ER. However, shared vision can help organizational members to minimize ambiguities and conflicting interests, thus underpinning the association between GTM and GOI and ER. We expect that the findings of this study will advance preliminary incongruous discoveries on the underlying linkage.

### Practical implications

The findings of this study provide meaningful insights for managers and service firms in the tourism industry. Given escalating gravity of embedding green practices across all business activities (Kalyar et al., 2021), service firms in the tourism industry require an imperative to develop and retain green talent to exploit pro-environmental strategies (Ogbeibu et al., 2021). As corroborated by Arshad et al. (2018), service firms in the tourism industry, in compliance with the UN’s sustainable development goals, face severe governmental pressures to comply with environmentalism, prompting service organizations to induce GTM strategies. Findings of this study endorse that GTM strategies translate into enhanced ER; thus, apposite organizational interventions are warranted to manoeuver GTM that result in developing and retaining green talent. To ensure human capital that manifests heightened environmental concerns, managers should nurture and retain green talent in organizations in order to cultivate environmental sustainability (Ogbeibu et al., 2021). Further, there is a great need to converge organizational (i.e., “GTM”) and employee levels (i.e., “talent perception,” and “retention”) objectives. Furthermore, the exclusivity of GTM strategy may gauge whether development initiatives are tailored to key people or positions or emphasize building more comprehensive organizational competence and social capital (Iles et al., 2010).

Second, our findings endorse that GOI offers a theoretically grounded explanation of *how* GTM predicts ER in the tourism industry. Managers can verify whether their firms’ GTM contributes to enhanced ER by observing their firms’ GOI. Although prior studies indicate that GTM could lead to turnover intention due to conflicting interests or ambiguities (Ogbeibu et al., 2021). Our findings indicate that GTM significantly affects ER through the sense of GOI. We thereby suggest managers to devise appropriate ways to foster GOI. In this milieu, Haslam et al. (2003) presented an all-inclusive model to nurture organizational identity. The authors proposed four phases of the development of organizational identity in their ASPIRe model of “Actualizing Social and Personal Identity Resources,” to inflate organizational consequences. The first phase refers to “detection of relevant identities” for employees in a given organizational unit. The second and third phase reflects goals linked with the respective identities with respect to the convergence of relevant subgroups and organizational unit. In the fourth phase, the newly developed organic identities transform into superior organizational planning and direction. Through instilling GOI, organizations can translate GTM into enhanced ER.

Last but not least, our findings stress the critical role of GSV as a boundary condition of the GTM-ER linkage. To implement GTM strategies effectively, organizations should inculcate environmentalism in their shared vision that managers and employees should concede. One way to implement GSV is by giving autonomy and freedom to members of an organization to address environmental challenges and concerns. In sum, organizations should establish a culture of GSV to strengthen the association between GTM, GOI and ER.

### Limitations and future research directions

The findings of this study should be trumpeted with its limitations. First, this study employed a time-lagged design to collect data from employees working in service firms in the tourism industry in Pakistan. In spite of that all the study variables were not tapped at all periods. Second, the findings of this study indicate that GOI partially mediates the relationship between GTM and ER. Therefore, we invite future studies to determine the TM-retention nexus through the mediator effect of other factors, *such as* green CSR (Wu et al., 2018) and psychological empowerment (Hartmann et al., 2018), etc. Third, the present study examines the boundary effect of GSV in the GTM-ER linkage. We suggest future studies assess the boundary effects of other individual and/or contextual factors to examine the contingent effect. Furthermore, we suggest a finer-grain investigation of the GSV construct from a multilevel perspective. For instance, future studies should assess *how* individuals and teams from different functional units enact GSV. Last but not the least, the findings of this study should not be generalized in Western countries due to the examination in the non-Western contexts, therefore, this merits further studies to be conducted in Western countries.

## Conclusion

The current study anchors on the SIT and SET to predict the association between GTM and ER through the mediator effect of GOI and moderator effect of GSV. In this milieu, the study investigates the boundary effects of individual as well as contextual factors that might influence the association between GTM and ER. Drawing on data collected from the service employees in the tourism firms, the analyses support all the study’s hypotheses. For instance, GTM significantly influence ER through the mediator effect of GOI. Further, GSV moderates the underlying linkage between GTM and ER, mediated by GOI such that at high levels of GSV the associations are more potent (and vice versa). The study presents unique and meaningful insights for theory and practice.

## Data availability statement

The raw data supporting the conclusions of this article will be made available by the authors, without undue reservation.

## Author contributions

All authors made a significant contribution to the work reported, whether that is in the conception, study design, execution, acquisition of data, analysis and interpretation, or in all these areas; took part in drafting, revising or critically reviewing the article; gave final approval of the version to be published; have agreed on the journal to which the article has been submitted; and agree to be accountable for all aspects of the work.

## Conflict of interest

The authors declare that the research was conducted in the absence of any commercial or financial relationships that could be construed as a potential conflict of interest.

## Publisher’s note

All claims expressed in this article are solely those of the authors and do not necessarily represent those of their affiliated organizations, or those of the publisher, the editors and the reviewers. Any product that may be evaluated in this article, or claim that may be made by its manufacturer, is not guaranteed or endorsed by the publisher.
