# Supplementary material for: Cultivating green workforce: The roles of green shared vision and green organizational identity
Source: Front Psychol. 2023 Mar 15;14:1041654. doi: 10.3389/fpsyg.2023.1041654 (PMC10050744; doi:10.3389/fpsyg.2023.1041654)
Supplement: Supplementary file 1 [file Data_Sheet_1.docx]

**Appendix I – Questionnaire**

Green talent management (GTM)

1. My organisation cares about my wellbeing and offers considerable support for my welfare when executing green centered initiatives.
2. My organisation offers green training, workshop opportunities, coaching and courses that advance my knowledge on how to foster environmental sustainability.
3. My organisation offers me a considerable degree of autonomy when carrying out green related tasks.
4. My organisation offers me job rotation opportunities associated with environmental sustainability.
5. My organisation is very supportive of green related activities that can help me plan my future development.
6. My organisation offers me challenging assignments that are grounded on environmental sustainability.
7. In my organisation, green tasks are driven with several opportunities that allow me express myself and share my opinions on green related matters.

Green organizational identity (GOI)

1. The company’s top managers, middle managers, and employees have a strong sense of the company’s history about environmental management and protection.
2. The company’s top managers, middle managers, and employees have a sense of pride in the company’s environmental goals and missions.
3. The company’s top managers, middle managers, and employees feel that the company has carved out a significant position with respect to environmental management and protection.
4. The company’s top managers, middle managers, and employees feel that the company have formulated a well-defined set of environmental goals and missions.
5. The company’s top managers, middle managers, and employees are knowledgeable about the company’s environmental traditions and cultures.
6. The company’s top managers, middle managers, and employees identify strongly with the company’s actions with respect to environmental management and protection.

Green shared vision (GSV)

1. There is commonality of purpose in my senior team.
2. There is total agreement on our organizational vision.
3. All senior team members are committed to the goals of this organization.
4. People are enthusiastic about the collective goals and mission of the whole organization.

Employee retention (ER)

1. I’m planning on working for another company within a period of three years.
2. Within this company my work gives me satisfaction.
3. If I wanted to do another job or function, I would look first at the possibilities within this company.
4. I see a future for myself within this company.
5. It doesn’t matter if I’m working for this company or another, as long as I have work.
6. If it were up to me, I will definitely be working for this company for the next five years.
7. If I could start over again, I would choose to work for another company.
8. If I received an attractive job offer from another company, I would take the job.
9. The work I’m doing is very important to me.
10. I love working for this company.
11. I have checked out a job in another company previously.
